# Pediatric Orbital Cellulitis Complicated by Cavernous Sinus Thrombosis: A Pediatric Simulation Case for Residents

**DOI:** 10.15766/mep_2374-8265.11633

**Published:** 2026-08-27

**Authors:** Jan Fune, Elana Siegel, Margaret Yang, Jennifer K Gillen, Jared M Kutzin

**Affiliations:** 1 Assistant Professor, Division of Pediatric Hospital Medicine, Department of Pediatrics, Mount Sinai Kravis Children's Hospital; 2 Fellow, Division of Pediatric Critical Care Medicine, Department of Pediatrics, Mount Sinai Kravis Children's Hospital; 3 Assistant Professor, Division of Pediatric Critical Care Medicine, Department of Pediatrics, Mount Sinai Kravis Children's Hospital; 4 Professor, Department of Emergency Medicine, Department of Medical Education, Icahn School of Medicine at Mount Sinai; Senior Director, Simulation, Teaching, and Research Center, Department of Emergency Medicine, The Mount Sinai Hospital

**Keywords:** Simulation, Pediatrics, Emergency Medicine, Pediatric Critical Care Medicine, Pediatric Emergency Medicine, Pediatric Infectious Diseases, Orbital Cellulitis, Cavernous Sinus Thrombosis

## Abstract

**Introduction:**

Orbital cellulitis is primarily a pediatric disease; although distinct from periorbital cellulitis, presenting symptoms may overlap, creating a challenge in diagnosis. If untreated, it can result in severe complications such as vision impairment, intracranial infection, cavernous sinus thrombosis (CST), and death. We created a simulation course to teach pediatric residents to recognize and treat this important diagnosis and its life-threatening complications.

**Methods:**

This simulation features a 10-year-old patient who presents with eye pain and swelling due to orbital cellulitis. Learning objectives include recognizing and managing orbital cellulitis, identifying sequelae of orbital cellulitis (eg, CST), and liaising with surgical and intensive care consultants. Facilitators led a postsimulation debrief and didactic session, and participants completed an anonymous postcourse evaluation. The entire session took 60 minutes.

**Results:**

This simulation was performed 4 different times, involving a total of 27 participants, all of whom were pediatric residents. Twenty-three participants completed the postcourse survey. The average overall course was rated 5.7/6, and participants reported that the knowledge gained from the session will be helpful to them in their practice and will help improve patient outcomes (mean = 3.9/4).

**Discussion:**

Our simulation was well received by participants and provided them with an opportunity to learn how to manage a highly morbid complication of orbital cellulitis, a common pediatric condition. This educational session can be completed in an hour and requires no advanced preparation for participants. This simulation can be adapted to run in a low-fidelity environment across a range of medical subspecialty training programs.

## Educational Objectives

By the end of this activity, learners will be able to:

1. Identify the signs and symptoms of orbital cellulitis, such as proptosis, ophthalmoplegia, and impaired vision.
2. Identify cavernous sinus thrombosis as a complication of orbital cellulitis.
3. Initiate appropriate imaging and antibiotics for management of cavernous sinus thrombosis.
4. Escalate to surgical and intensive care subspecialists to transition care.

## Introduction

Orbital cellulitis is a life-threatening infection of the contents of the ocular orbit, including the fat and extraocular muscles. It can be confused with periorbital cellulitis, as both present with ocular pain, eyelid swelling, and erythema.^1^ Whereas periorbital cellulitis is typically a mild condition arising as a sequela from insect bites or eyelid trauma and confined to the superficial periocular tissues, orbital cellulitis is a sight and life-threatening entity, arising almost always as a complication of sinusitis.^2^ If not treated emergently, it can lead to vision loss and fatal complications caused by morbid sequelae of intracranial extension of the infection, manifesting as meningitis, sepsis, brain abscess, septic emboli, and cavernous sinus thrombosis (CST).^3^ It is primarily a disease of children, with an incidence of 1.6 per 100,000 children versus 0.1 per 100,000 of adults,^4^ making it an important topic of clinical review for providers who care for children. As the vaccine landscape evolves across the United States, previously rare diagnoses caused by vaccine-preventable illnesses may see a resurgence, such as orbital cellulitis due to *Hemophilus influenzae*.^5^ Thus, medical providers should be familiar with its clinical presentation, as well as its serious complications. Neurologic involvement should be suspected when systemic features are present, such as headache, vomiting, dizziness, altered consciousness, hemiparesis, speech and/or vision disturbances, or seizure, and should be addressed emergently to prevent further deterioration.^6^ This clinical presentation should trigger aggressive antibiotic management, as well as neuroimaging and surgical consultation. Patients with orbital cellulitis are at risk for developing CST and stroke due to these neuroinfectious complications^7^; any clinical change suspicious for stroke should prompt rapid escalation of patient care.^8^

In January 2020, our institution instituted a new process for obtaining rapid consultations from the pediatric intensive care unit (PICU) team and initiating PICU transfers. Around this time, there was an incident where an admitted patient admitted for orbital cellulitis developed neurologic symptoms that were nearly missed, and the patient ultimately required PICU-level care. This provided us with an opportunity to create a simulation that would teach residents how to identify and manage a rare but serious sequela of a common pediatric diagnosis and allow them to practice escalating patient care to the PICU. We chose to develop a simulation because it is particularly well suited to this topic, as it represents a high-acuity, low-frequency clinical scenario where real-world exposure is limited.^9^ More so, experiential learning through simulation allows learners to practice time-sensitive decision-making, clinical reassessment, and escalation of care in a psychologically safe environment. No resources were identified within *MedEdPORTAL* related to pediatric orbital cellulitis and its potential complications. We reviewed *MedEdPORTAL* publications that focused on pediatric cases to develop and structure our simulation,^10-13^ and we sought to create a simulation geared toward pediatric residents at all levels of training. The primary focus of our simulation is the recognition of orbital cellulitis and its complications, with emphasis on identifying neurologic deterioration and appropriately escalating care.

## Methods

### Development

A group of physicians with experience in simulation and curriculum creation formed the development team. The scenario (Appendix A) was based on a common pediatric illness, and was written by a pediatric hospitalist, a pediatric hospitalist with pediatric emergency medicine experience, a second-year PICU fellow, and a PICU attending. The director of the Simulation Teaching and Research (STAR) center at our institution reviewed the case for its feasibility and applicability to typical simulation frameworks. The director of the simulation center also trained the other group members on orienting the participants to the simulation center and debriefing using the Promoting Excellence and Reflective Learning in Simulation (PEARLS) framework.^14^ All group members reviewed the simulation case to ensure its accuracy and appropriateness for pediatric residents at all levels of training. Subsequently, the case was piloted by a group of 3 trainees consisting of a second-year Pediatric Hospital Medicine fellow, a PGY-4 pediatric chief resident, and a PGY-2 pediatric resident to ensure the difficulty level was appropriate for our program's pediatric residents and to identify areas for improvement. The pilot revealed technical issues, such as the trainees not hearing the patient moaning in pain or slurring words as the case progressed. We addressed this before implementing this scenario during the pediatric residency's simulation curriculum by training the simulation technician. Notably, no prerequisite knowledge or reading was required for the pilot run or the final version of the simulation encounter. This project was considered exempt by the Icahn School of Medicine at Mount Sinai Institutional Review Board (IRB-20-04151/HSM 20-01201).

### Equipment/Environment

The simulation was conducted in our simulation center using a high-fidelity manikin, though it could be performed in an actual patient bed (eg, emergency department or pediatric unit) in hospitals without access to a simulation lab. We used 4 x 4 gauze to make the left eye appear swollen by stuffing it underneath the manikin's left eyelid, and red eyeshadow was used to make the eyelid appear erythematous. Participants were advised to ask the facilitators to clarify or confirm any exam findings during the simulation, especially if they were unsure of their assessments of a manikin.

A photo of the manikin's face, equipment, and medications used in this simulation are detailed in Appendix B. The scenario began with the parent at the patient's bedside; the patient did not have IV access and was not connected to any monitoring. The facilitator portraying the nurse was prepared to provide the trainees with simulated medications and IV fluids. Results of the workup appeared on a monitor when requested by the simulation participants (Appendix C). Trainees could call and speak to consultants using a phone that connected them to 1 of the facilitators, who portrayed the consultants.

In Appendix B, we provide suggestions on how to simulate the patient for those who may not have access to, or choose not to use, a high-fidelity manikin or a simulation technologist. Regardless of the resources or technology available, we suggest that facilitators encourage participants to suspend disbelief and to ask clarifying questions as they arise during the encounter, especially if the exam changes appear subtle or difficult to simulate. In these instances, facilitators could point out exam changes or endorse that the patient is complaining of blurry vision, or that the patient is not moving 1 side of the body. For example, ophthalmoplegia was conveyed by stating that the patient could not move the affected eye; cranial nerve deficits were described verbally; and unilateral weakness was communicated as decreased movement on 1 side.

### Personnel

Required personnel include at least 1 facilitator to orient the trainees to the simulated scenario, provide diagnostic test results, and lead the debrief session. Additional personnel would ideally include an additional facilitator to perform any of the following roles: nurse, consultant, parent, or co-instructor. The team could also benefit from a simulation technologist (if available) to update vital signs and physical exam findings in response to interventions. If personnel members are limited, roles can be combined: for instance, the same facilitator could act as the instructor, debriefer, and nurse, or the simulation could be performed without the parent if a facilitator can provide the clinical history.

Each of our sessions accommodated 4 to 7 participants. Based on our observation, the teams divided themselves into the following roles (described next). However, if there are fewer than 4 participants, roles can be combined (eg, 1 team member can perform the physical exam and act as the family correspondent).

Team members could potentially include any of the following:

• Participant #1: Team leader
• Participant #2: Airway physician
• Participant #3: Survey/physical exam physician
• Participant #4: Family correspondent/historian
• Participant #5: Liaison with consultants
• Participant #6: Timekeeper/recorder

### Implementation

The simulation was implemented as part of the pediatric residency's simulation curriculum. Each simulation group was allotted 1 hour to orient participants to the simulation center and equipment, perform the simulated encounter (see Appendix A), debrief (Appendix D), review didactic slides (Appendix E), and complete a course evaluation form (Appendix F). An example timeline is described next:

• Orientation to the simulation encounter: 5 minutes
• Simulation case: 20 minutes
• Debrief: 15 minutes
• Didactic slides: 15 minutes
• Course evaluation: 5 minutes

Upon arrival at the simulation center, learners were oriented and facilitators reviewed the manikin's capabilities (eg, “You can feel femoral pulses on this manikin”). After the orientation, the facilitator called the team into the emergency department room to evaluate a 10-year-old patient brought in by a parent for fever and worsening left eye swelling (the scenario is described in detail in Appendix A). Before participants entered the room, facilitators reminded them that simulations generally run on an accelerated timeline.

The case began as soon as the participants entered the room. On initial assessment, participants saw an uncomfortable-appearing patient with notable left eye swelling and surrounding erythema.

Subsequently, the team asked the nurse to connect the patient to the monitor (or the trainee set up the monitor independently). The parent provided a brief history and only provided additional information upon request. Once learners identified that the patient had signs and symptoms concerning for orbital cellulitis, they initiated IV antimicrobials and requested labs. Despite these appropriate interventions, the patient went on to develop ophthalmoplegia, cranial nerve palsy, and sudden onset unilateral weakness. These new findings prompted the team to consider intracranial extension and obtain a CT scan to look for complications of orbital cellulitis. The CT scan confirmed left orbital cellulitis and raised the concern for CST. The scenario concluded when 1 of the participants spoke with a surgical consultant (eg, ear, nose and throat; or neurosurgery) and gave an appropriate handoff to the PICU. Afterward, learners were brought out of the simulated patient room for a facilitated debrief (see Appendix D) followed by didactic slides (see Appendix E) and a course evaluation (see Appendix F).

### Debriefing

All facilitators participated in the debriefing session, though 1 facilitator was designated to lead the discussion and ensure we followed the PEARLS framework (see Appendix D).

Per the PEARLS framework, the debriefing session should begin with an open-ended question such as “How are you feeling?” to gauge and respond to the learners’ emotions. After emotions are addressed, the facilitator(s) should ensure that all learners understand the clinical course of the simulation and are able to identify the main issues they faced. This part of the debrief also provided an opportunity to remind participants that the time frame was greatly accelerated for the purpose of the simulation. Once this is complete, the facilitator moves to the analysis phase and, finally, the application and summary phase. In our debriefs, we invited each learner to share 1 thing they learned from this simulation that they hope to retain. Once this step was completed, the facilitators addressed knowledge gaps identified during the simulation or the debrief and concluded with a didactic PowerPoint on orbital cellulitis (see Appendix E).

### Assessment

We created an anticipated list of actions we hoped the participants would perform during the encounter (Appendix G) based on consensus within our simulation group. We piloted the checklist with our pilot group (as described in the Methods section) and revised it to ensure it captured the key elements. This checklist helped facilitators track the actions completed by the participants during the simulation, which helped guide the postsimulation debrief since facilitators could highlight actions that were performed successfully, partially, or not at all. This checklist included space for facilitators to take notes, which could be referenced and updated throughout the debrief as participants shared their reflections through each phase of the PEARLS framework.

At the end of the didactic session, the learners voluntarily completed an electronic course evaluation (see Appendix F). Notably, the course evaluation we used was the standard evaluation form used for all simulation-based education programs in our program, which assesses Kirkpatrick level 1.^15^ The evaluation comprised of Likert scale questions and an open-ended question (“My biggest take home from this course was…”); it asked the learners to evaluate the overall quality of the course. Questions using the Likert scale included:

1. How realistic did you feel the simulation experience was?
2. The debriefing session enhanced my knowledge.
3. The knowledge I gained from the session will be helpful to me in my practice
4. What I learned today will help to improve patient outcomes.

Free-text responses were reviewed by the authors and are presented as representative quotes aligned with the learning objectives.

## Results

After the pilot, this simulation was completed 4 separate times by 4 different groups of pediatric residents from the same training program between April 2024 and December 2024. Each simulation was led by the same group of facilitators: 2 pediatric hospitalists, 1 PICU fellow, and the director of the simulation center; each facilitator had a defined role during the course (eg, manikin operator and lead facilitator for the debriefing session). The facilitators arrived at the simulation center 30 minutes before the scheduled simulation to divide roles and ensure all equipment was working correctly.

On average, each group completed the simulation within 12:06 minutes (min = 11:06, max = 14:25). Twenty-seven pediatric residents completed the simulation, and 23 voluntarily completed the course evaluation. The respondents included 7 PGY-1s, 6 PGY-2s, and 10 PGY-3s. During the “description” phase of the group debrief, participants described and discussed the signs and symptoms of orbital cellulitis, their concern for neurologic involvement as the case evolved, and why they sought consultant recommendations and PICU-level care. In the analysis phase, participants shared what they felt they managed well and what they would have done differently. Elements of Kirkpatrick's level 2 of learning were thus clarified during the simulation debrief, when discussing the key learning points of the case.

Results of the course evaluation (Table) captured their reactions and indicated that the participants felt the simulation was realistic (mean = 5.1/6) and that the debriefing session enhanced their knowledge (mean = 3.9/4). The evaluation results also suggested that the knowledge gained from the session will be helpful to them in their future practice and will help improve patient outcomes (mean = 3.9/4). The average overall course rating was 5.7 on a scale of 1 (poor) to 6 (excellent).

**Table.**
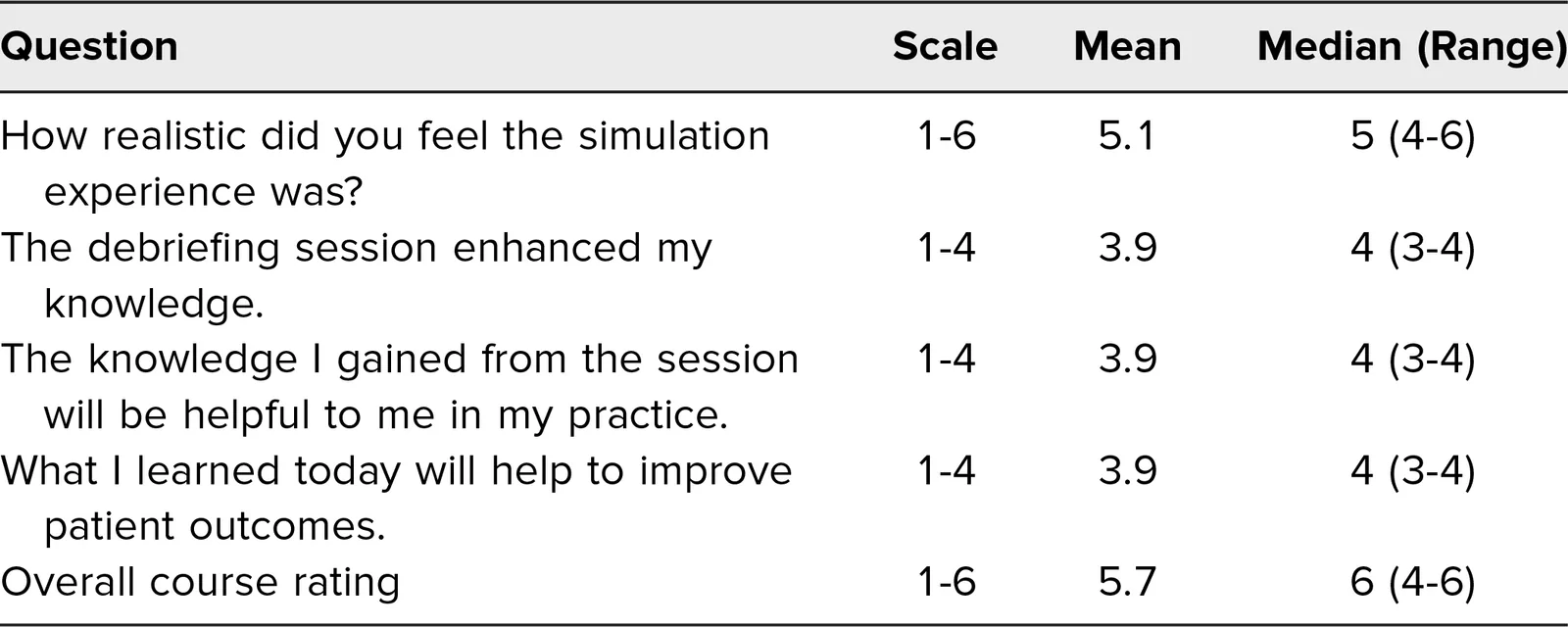
Course Evaluation Scores

We reviewed the free-text responses to the following question: “My biggest take home from this course was....” Responses related to our learning objectives included “cavernous sinus thrombosis is an uncommon complication of orbital cellulitis” and “how to manage acute sequela of orbital cellulitis.” Other take home messages pertained to defining roles, such as “how important it is to have defined roles, say them out loud, and do frequent recaps” and “not to split my attention as code leader – important information can be missed if giving orders and trying to listen to team members at the same time.” Additional quotes include “Continue management until consultants arrive" and “Keep venous thrombosis higher on differential.”

## Discussion

This simulation-based curriculum provided learners with an opportunity to recognize and act on a rare but critical sequela of orbital cellulitis (a common pediatric condition), practice teamwork and communication skills, work with consultants, and escalate care. Additionally, this case contributes to the growing body of published pediatric simulation cases on *MedEdPORTAL.* In addition to the detailed simulation case, we provide several items that would allow educators to reproduce this course at other institutions, such as the debriefing script, didactic slides, and evaluation. Notably, learners do not need prerequisite knowledge to participate, nor do they need to do any preparatory reading.

Piloting the simulation and soliciting feedback with a group of more senior learners helped us improve the flow of the simulated scenario before officially incorporating it into the pediatric residency's simulation curriculum. We recommend piloting the case before running it with learners to elicit feedback that may help with implementation at users’ specific institutions. This may be especially helpful to practice applying the case in a lower-fidelity setting or with a different learner type (eg nursing students or emergency room providers). For future iterations, we recommend that facilitators familiarize themselves with debriefing techniques. Importantly, the facilitator leading the debrief should be familiar with the PEARLS framework. Facilitators can prepare by reading the PEARLS paper by Eppich and Cheng,^14^ which describes each phase of the framework in detail, and they can refer to Appendix D, which includes sample questions and responses. This became particularly salient with 1 group of participants: when asked “How are you feeling?” they launched straight into talking about how to improve their future performance; this required careful moderation by the facilitator to adhere to the stepwise PEARLS approach. Our anticipated action list provided direction in moving into the analysis portion of the framework.

Our institution is privileged to have access to a high-fidelity simulation lab. However, we recognize that this is not widespread. The simulation could be run in a lower-fidelity setting, whereby a designated simulation facilitator can play the role of “technology”: calling out vital signs and physical exam changes as they occur, and/or responding to concrete questions by the participants (eg “Do the patient's pupils constrict to light”?). Similarly, the facilitator playing the role of the parent or nurse can support the simulation by calling out relevant physical exam observations (eg, “My son sounds funny” or “Why does my son's eye look like that?”). As with most simulations, we advise the facilitator to encourage participants to suspend disbelief during the encounter.

Overall, the course was very well received by all participants, as reflected in the overall course rating and the verbal feedback shared with us at the session's conclusion. In addition to our course evaluation results, we learned that we met our learning objectives during the application/summary phase of the PEARLS debrief. Learners shared reflections and insights related to our objectives, including keeping CST higher on their differential for patients with complications of orbital cellulitis, and remembering to reassess vital signs and physical exam findings after different interventions.

### Limitations

Our study is limited by the lack of survey results from 4 of the participants, which may have affected the final evaluation score. This study is also limited by the group sizes being inconsistent (groups ranged from 4 to 7 participants). While we learned from our pilot session that although the case could be performed with smaller sizes, the smaller groups may have been disadvantaged, as there were fewer people to play all possible roles in the simulation, making for variable experiences across our participant groups. In the future, we recommend standardizing the group sizes. “Lack of role assignment” was a common response in the self-evaluation; future simulations could incorporate the use of a communication checklist or facilitators can help encourage teams to define roles before they enter the encounter. We also recognize that not all institutions have subspecialty consultants readily available. In those instances, the simulation can end once the participants obtain head imaging, and facilitators can describe the abnormalities seen on head CT. Lastly, we evaluated lower levels of the Kirkpatrick hierarchy but did not formally evaluate participants’ knowledge. It is difficult to assess whether this simulation led to improved patient outcomes, as CST is a rare event, which is why we chose to teach its management through simulation. However, learners shared what they learned during the application/summary phase of the debrief (“Please share one take-away that will help you next time you have a patient with orbital cellulitis and stroke-like symptoms”).

This simulation, which focused on a rare but dangerous complication of a common pediatric illness, exposed learners to a clinical scenario that requires rapid diagnosis, effective communication, and timely management. Our findings show that this 60-minute session can be impactful in teaching residents how to identify and treat complications of orbital cellulitis. For future iterations, we will obtain more objective data on the effect of this simulation on participants, such as performing a pre- and postsimulation survey to measure knowledge gained about the management of orbital cellulitis.

## Appendices


Simulation Case.docxEquipment List.docxLab and CT Scan Results.pptxSimulation Debrief.docxDidactic Slides.pptxCourse Evaluation.docxAnticipated Actions.docx
*All appendices are peer reviewed as integral parts of the Original Publication.*
