# Supplementary material for: Pediatric Orbital Cellulitis Complicated by Cavernous Sinus Thrombosis: A Pediatric Simulation Case for Residents
Source: MedEdPORTAL. 2026 Aug 27;22:11633. doi: 10.15766/mep_2374-8265.11633 (PMC13518600; doi:10.15766/mep_2374-8265.11633)
Supplement: Supplementary file 1 [file mep_2374-8265.11633-s001.zip › A. Simulation Case.docx]

| **Appendix A: *MedEdPORTAL* Simulation Case Template**    **SIMULATION CASE TITLE: Pediatric Orbital Cellulitis Complicated by Cavernous Sinus Thrombosis**: **A Pediatric Simulation Case for Residents**    **AUTHORS: Jan Fune, MD, Elana Siegel, MD, Margaret Yang, MD, Jennifer K Gillen, MD, Jared M Kutzin, DNP, MS, MPH, RN, FSSH, FAAN**    **LEARNER AUDIENCE: Pediatric Residents (PGY 1-3)** | |
| --- | --- |
| **PATIENT NAME: Oliver O’Connor**    **PATIENT AGE: 10-years-old**    **CHIEF COMPLAINT: Left Eye Swelling and Pain**    **PHYSICAL SETTING: Pediatric Emergency Department** | |
| **if if** | |
| **Brief narrative description of case** | A 10-year-old male presents to the emergency department for left eye swelling and ocular pain. Upon arrival to the ED, he is noted to be febrile, tachycardic, and uncomfortable-appearing.    Learners will begin diagnostic evaluation and management with laboratory testing and initiating empiric antimicrobials after identifying orbital cellulitis. Despite appropriate interventions, the patient will decompensate and develop worsening eye pain and sudden-onset ophthalmoplegia. The team should note these clinical changes, implement critical measures, activate pediatric critical care and surgical subspecialty consultation, and urgently obtain head imaging while expanding antimicrobial coverage. CT imaging reveals orbital cellulitis complicated by cavernous sinus thrombosis. Pediatric Intensive Care Unit (PICU) and surgical subspecialty consultations (e.g., Neurosurgery, Ophthalmology, or ENT) should be obtained. If head imaging is not obtained, or the abnormal findings are not identified, the patient will continue to deteriorate and eventually seize.    Throughout the case, participants will need to continually reassess the patient. The case is concluded upon completion of neurologic imaging and escalating care to the surgical and intensive care teams.  Note: the time frames in this case are accelerated due to the simulated environment |
| **Primary Learning Objectives** | By the end of the simulation, learners should be able to:   1. Identify the signs and symptoms of orbital cellulitis, such as proptosis, ophthalmoplegia, and impaired vision 2. Identify cavernous sinus thrombosis (CST) as a complication of orbital cellulitis 3. Initiate appropriate imaging and antibiotics for management of CST 4. Escalate to surgical and intensive care subspecialists to transition care |
| **Critical Actions** | 1^st^ Clinical State: Presentation   1. Identify orbital cellulitis 2. Initiate goal directed therapy, which should include obtaining diagnostic labs (e.g., blood culture) and initiating empiric antimicrobials 3. Order anti-pyretic(s) 4. Use closed loop communication with all team members     2^nd^ Clinical State: Decompensation   1. Identify acute focal neurological changes: cranial nerve changes, double vision, and slurred speech 2. Perform a repeat focused neurologic exam 3. Order head imaging to evaluate neurologic findings 4. Consult pediatric surgery specialty (e.g., Neurosurgery, Ophthalmology, or ENT) or PICU 5. Escalate the level of care and provide warm hand off to PICU 6. Use closed-loop communication with all team members |
| **Learner Preparation or Prework** | The learners will be oriented to the simulation room and given a brief introduction to the case before commencing. |

| **Initial Presentation** | | | |
| --- | --- | --- | --- |
| **Initial vital signs** | T: 39.6 C, HR: 146, RR: 30, BP: 110/70, SpO2: 99%, Weight: 30 kg | | |
| **Overall Setting and Appearance** | Learners will enter the emergency department room and see a child in moderate discomfort, lying supine on the gurney. His parent is trying to console him. The lights are off or dim in the room. | | |
| **Standardized Participants (and their roles in the room at case start**) | The following roles can be combined based on the personnel available. At least 1 facilitator is required to conduct this simulation. We provide suggestions for how to assign and separate optional roles for each facilitator below:  Facilitator #1: Simulation instructor and debriefer; will verbally provide specific physical exam findings if asked by the learners    Facilitator #2: Can play the role of parent and/or consultant; assists with debrief  Bedside nurse (RN): to obtain IV access, draw labs, dispense medication  Parent: to describe HPI and medical background of patient (may be played by facilitator/instructor); can also help redirect team if needed (e.g., “why does his left eye look worse now?”)  Simulation technologist: Ensures high fidelity simulation by changing vital signs and physical exam findings based on interventions (this role may only apply if a high fidelity Manikin is being used)    Team members could potentially include any of the following**:**  Participant #1: Team leader  Participant #2: Airway physician  Participant #3: Survey/physical exam physician  Participant #4: Family correspondent/historian  Participant #5: Communicator with consulting teams | | |
| **HPI** | Volunteered information:  10-year-old male “Oliver” presents to the emergency department, brought in by his mother for 2 days of left eye swelling. He developed a runny nose about 4 days ago, with nasal congestion and a dry cough. Then his left eye became a little puffy 2 days ago; this has worsened, and now he can’t open his eye spontaneously anymore and has started complaining of a headache. Last night, he started having some greenish discharge from the affected eye, prompting ED presentation.  Information that must be asked:  S: HPI as above  A: allergic to sulfa drugs (breaks out in hives)  M: no medications  P: does not wear glasses or contacts; gets seasonal allergies; no surgical history  L: last oral intake 4 hours ago  E: no recent trauma to the eye(s), recently attended day camp in a wooded area; no known sick contacts | | |
| **Past Medical/Surgical History** | **Medications** | **Allergies** | **Family History** |
|  | None | Sulfa medications | Mother with history of atopy, eczema, and asthma |
| **Physical Examination** | | | |
| **General** | No acute distress but appears uncomfortable | | |
| **HEENT** | Left eye with upper and lower lid swelling, warmth, erythema, induration, no proptosis, and scant clear discharge. Pupils 4mm and reactive, symmetric. EOM intact but reporting some discomfort with movements. Right eye exam is WNL. | | |
| **Neck** | Normal range of motion, no neck stiffness, no palpable lymph nodes | | |
| **Lungs** | Clear to auscultation bilaterally, good air entry | | |
| **Cardiovascular** | Regular rate and rhythm, tachycardic, no murmurs, capillary refill less than 2 seconds | | |
| **Abdomen** | Soft, non-tender, no masses | | |
| **Neurological** | GCS is 15, awake, alert, normal strength and tone, cranial nerves 2 – 12 are intact; negative Kernig and Brudzinski signs | | |
| **Skin** | No petechiae or purpura | | |
| **GU** | Circumcised male, no rashes or lesions | | |

| **Instructor Notes - Changes and CASE Branch Points** | | |
| --- | --- | --- |
| **Intervention / Time point** | **Change in Case** | **Additional Information** |
| Learners verbalize a differential diagnosis, including orbital cellulitis  T = 4 minutes (00:04) | Exam as above. Learners should:   - Establish their roles - Assess ABCs - Obtain vitals and/or place patient on monitor - Initiate goal directed therapy - Order basic labs: CBC, BMP, blood culture, CRP and/or procalcitonin - Obtain IV access - Order anti-pyretic - Order 20 mL/kg of isotonic fluids | If learners do not give IV fluid bolus and/or antibiotics, patient’s heart rate will **elevate to 160** |
| T = 1 minutes (00:05)    Learners ask for lab results |  | VBG: 7.36/39/72/19 lactate 2.2  POCT glucose 88  Remainder of blood work is ‘pending’    If asked for comprehensive VBG results, hematocrit 30, sodium 136, potassium 3.6 |
| T = 2 minutes (00:07)     - Learners request new vital signs - Learners request new neurologic exam | Patient begins to cry out that his headache is much worse, sharp. His eye is hurting even worse and he can’t see well.    PE: left eye ptosis and chemosis, 7 mm dilated, lateral gaze palsy (unable to abduct left eye), mild exophthalmos. Mild right eyelid swelling and chemosis.  Patient is drowsy but arousable.    T: 37.5 C, HR 114, RR 26, BP 109/77, SpO2 99%, | If learners miss this cue from the patient, mom or nurse state that his eye looks "funny” and he is seeing double.    If the learners continue to miss the cranial nerve changes, he will develop total nerve palsy (eye is down and out) with worsening exophthalmos, overall mental status, and finally seizure. |
| T = 2 minutes (00:09)     - Learners request new vital signs - Learners request updated and full neurologic exam | Neuro exam: AAOx1 (name only). No neck stiffness, no meningismus, no photophobia. | Patient continues to act confused with the aforementioned ocular changes. |
| T = 2 minutes (00:11)   - Airway should be reassessed | Patient is protecting airway, SpO2 99%, lungs CTAB, intact gag reflex | If learners do not recognize symptoms of CST, the patient will begin having slurred speech |
| T =  4 minutes (00:15)    - Learners should make patient NPO - Learners call the ICU | If patient begins seizing, learners should administer IV lorazepam (0.05 – 0.1 mg/kg/dose) | If learners do not discuss escalating care and alerting the ICU, the patient will start seizing    Learners should use IV access to give anti-epileptic medication if needed (rather than rectal diazepam) |
| T = 2 minutes (00:17)    If not initially started, learners broaden antimicrobials to:   - Ceftriaxone (add 50mg/kg to make up meningitic dosing) - Vancomycin 15mg/kg - +/- metronidazole | Patient remains stable for CT scan | If learners order any oral medications, nurse should ask “is it safe for him to swallow medications given his altered mental status?” Learners should then make patient “NPO”.      Nurse will ask for what doses of antimicrobials    Ceftriaxone should be ordered at 100 mg/kg (meningitic dosing) |
| T = 4 minutes (00:21)    Learners order two CTs:     - CT head **with or without** contrast - CT orbits **with** contrast (to look for abscess) | Patient is stable for CT and is cooperative for study | Nurse to ask learners if the patient should or should not receive IV contrast.    Nurse to ask learners if priority is to give the contrast or antimicrobials through the IV.    Learners should attempt for 2^nd^ IV access if not already obtained so both can be given.    Patient will continue to decompensate, and eventually start seizing, as part of the disease course. Administering anti-epileptic medications, starting empiric antimicrobial therapy for presumed meningitis, and obtaining head imaging are expected responses to this clinical change. |
| T = 5 minutes (00:26)     - Learners call radiology to ask for the CT findings - Learners identify left orbital cellulitis and concern for intracranial process | Learners should explain diagnosis and next steps to parents | If learners do not call radiology, nurse should prompt them to call radiology for CT results  Despite ongoing management, the patient continues to deteriorate as part of the disease course. Consultation and escalation are expected responses to clinical deterioration. |
| T = 4 minutes (00:30)  Learners consult surgical subspecialty (either Neurosurgery, Ophthalmology, or ENT) and/or call PICU | Surgical subspecialty resident or PICU Rapid Response Team available and requests handoff. In places without subspecialty consults, participants can simulate calling a tertiary care center to request transfer to their institution and provide a warm hand off to the accepting physician.    Example hand off:    “This is a 10-year-old boy with fever and left eye pain and swelling concerning for left orbital cellulitis with physical exam and CT findings concerning for cavernous sinus thrombosis. He has received one 20mL/kg NS bolus, IV ceftriaxone and vancomycin. Currently afebrile, hemodynamically stable with left 6^th^ cranial nerve palsy and right-sided weakness. We’re calling you now for potential surgical intervention and ICU monitoring.” | Learners should use closed loop communication and demonstrate adequate handoff skills    Learners could consider anti-epileptic medications, anticoagulation, and MRV imaging  (though they do not need to initiate any of these)    Learners should use closed loop communication and SBAR |

**Ideal Scenario Flow**

The learners enter the ED and find a 10-year-old male patient who appears uncomfortable but not in distress. They recognize the patient has left eye swelling and notice the patient is febrile but otherwise hemodynamically stable. Learners should recognize the diagnosis of orbital cellulitis and begin obtaining history while simultaneously ordering a 20 mL/kg bolus of isotonic fluids, an anti-pyretic, a CBC and blood cultures prior to starting empiric antimicrobials. Despite appropriate management thus far, the patient will begin to develop ophthalmoplegia and cranial nerve palsy, concerning for a cavernous venous thrombosis. This should prompt learners to urgently request CT imaging given new focal findings. Learners should continuously monitor vital signs throughout the case and reassess his mental status, airway, and neurologic exam. Learners should broaden antimicrobial coverage for intracranial infection (if oral antimicrobials were initially started, they should be converted to IV forms). Learners will call consultant(s) throughout the simulation, but they should continue to manage the patient until they arrive. The case ends after speaking with a surgical team and providing verbal hand off to them and the PICU. Learners should demonstrate closed loop communication throughout the case.

**Anticipated Management Mistakes**

We recommend that facilitators make themselves familiar with this section as these anticipated mistakes may occur and will need to be discussed during the debriefing session.

1. *Failure to recognize mental status change and signs of cavernous sinus thrombosis: The patient is having ophthalmoplegia with acute changes in his cranial nerves, along with drowsiness and confusion, concerning for cavernous sinus thrombosis. If these worrisome changes are not recognized, the patient will develop a worsening neurological status, lose his airway, and begin seizing to make signs of CST more obvious. Learners may not realize that the manikin’s voice was slurring and may need prompting to notice this change.*
2. *Failure to order imaging: Participants should order images to help confirm the diagnosis of orbital cellulitis complicated by CST. If they do not, the consulting services they contact can prompt them to order the correct images.*
3. *Failure to institute critical care support measures in the setting of acute decompensation: The participants should institute continuous monitoring and airway monitoring, obtain a second IV access, escalate to the PICU, and alert neurosurgery, ENT, or ophthalmology.* *In places without subspecialty consults, participants can simulate calling a tertiary care center to request escalation of care.*
