# Supplementary material for: Pediatric Orbital Cellulitis Complicated by Cavernous Sinus Thrombosis: A Pediatric Simulation Case for Residents
Source: MedEdPORTAL. 2026 Aug 27;22:11633. doi: 10.15766/mep_2374-8265.11633 (PMC13518600; doi:10.15766/mep_2374-8265.11633)
Supplement: Supplementary file 1 [file mep_2374-8265.11633-s001.zip › B. Equipment List.docx]

**Appendix B. Equipment List**

- Manikin
- 4 x 4 gauze or any soft matter (e.g., tissue or cotton ball) to stuff under the left eyelid to make it appear swollen
- Makeup or red marker to make the left eye appear red
- Vital sign monitor
- Monitor to display results (e.g., imaging results)
- Phone (to call consultants, PICU, and/or stroke code)
- Stethoscope
- Oxygen
- Nasal cannula
- Simple face mask or non-rebreather mask
- Bag-valve-mask
- IV kit
- Medications available:
  - Normal saline
  - Acetaminophen (IV or oral)
  - Ibuprofen (or IV Toradol)
  - Ceftriaxone
  - Vancomycin
  - Metronidazole
  - Hypertonic saline
  - IV lorazepam
  - Mannitol

Note: If a monitor is not available, please print the lab results and images found in Appendix C

Note: To simulate cranial nerve palsy, we suggest having the left eye look downward and outward, and the pupil should be dilated. If this cannot be done, the facilitator(s) can inform participants that the patient is complaining of blurry vision. For unilateral weakness, we suggest that you have one side of the body have spontaneous movement. This may be a subtle exam change, so facilitator(s) can point out to participants that one side of the body has decreased movement. For example, ophthalmoplegia can be communicated by stating “the patient is unable to move the affected eye”, and unilateral weakness can be communicated by “the patient is not moving one side of his body.”

Examples of Manikin’s face to demonstrate left orbital cellulitis (These images are author owned) are shared below, in the following pages. Please note the left eye is red; we stuffed gauze under the eyelid to make it look swollen.

Example of Manikin’s face to demonstrate left orbital cellulitis (This image is author owned)

Example of Manikin’s face to demonstrate left orbital cellulitis (This image is author owned)

Example of Manikin’s face to demonstrate left orbital cellulitis (This image is author owned)
