# Supplementary material for: Pediatric Orbital Cellulitis Complicated by Cavernous Sinus Thrombosis: A Pediatric Simulation Case for Residents
Source: MedEdPORTAL. 2026 Aug 27;22:11633. doi: 10.15766/mep_2374-8265.11633 (PMC13518600; doi:10.15766/mep_2374-8265.11633)
Supplement: Supplementary file 1 [file mep_2374-8265.11633-s001.zip › D. Simulation Debrief.docx]

**Appendix D. Simulation Debrief**

Upon completion of the simulated scenario, facilitators should invite the learners to reflect using a scripted debriefing script. We modeled our debriefing session using the Promoting Excellence and Reflective Learning in Simulation (PEARLS) framework (as seen below).^1^ The session should begin by “setting the stage” for debriefing by ensuring a safe learning environment and then move onto the following phases in sequential order: reactions, description, analysis, and application/summary.

| Phase | What you might say to promote reflection |
| --- | --- |
| Reaction  Respond to learners’ emotions | - How are you feeling? - How are the rest of you feeling? - Other reactions? |
| Description  Make sure everyone is on the same page | - Can someone summarize the case from a medical point of view so that we are all on the same page? - From your perspective, what were the main issues you had to deal with?   *Potential follow up questions:*   - What happened next? - What things did you do for the patient? |
| Analysis  Promote reflection on performance | - Now that we are clear about what happened, let’s talk more about that case. - What aspects of the case do you think you managed well and why? - What aspects of the case would you have done differently and why? - I noticed you [*behavior*]. *After, describe a desired behavior or explore why an action was done.*   - Next time, you may want to…[*suggested behavior*]…because [*provide rationale*].’’     - “I noticed you say we should give the patient a bolus of fluids, but the patient did not receive it. Next time, you may want to assign a specific person to start a bolus and ask them to use closed-loop communication to make sure they were given.”   - I would like to understand why that felt like the right choice at that moment. - How would you describe how you worked as a team? |
| Application/ Summary  Review key learning points | - Please share one take-away that will help you next time you have a patient with orbital cellulitis and stroke-like symptoms - In summary, this was a case of a patient who presented with fever, eye pain, and headache, consistent with orbital cellulitis. He then developed symptoms concerning for stroke secondary to cavernous sinus thrombosis. - The key learning points from this case were (can transition to prepared PowerPoint, Appendix, at this point of the debrief if needed)   - Orbital cellulitis can have several complications, though uncommon, including some that are life threatening such as cavernous sinus thrombosis   - Cavernous sinus thrombosis can present with focal neurologic symptoms due to a venous infarction, and should prompt multidisciplinary stroke evaluation and management |

**References:**

1. Eppich W, Cheng A. Promoting Excellence and Reflective Learning in Simulation (PEARLS): development and rationale for a blended approach to health care simulation debriefing. *Simul Healthc*. 2015;10(2):106-115. doi:10.1097/SIH.0000000000000072
