# Supplementary material for: Pediatric Orbital Cellulitis Complicated by Cavernous Sinus Thrombosis: A Pediatric Simulation Case for Residents
Source: MedEdPORTAL. 2026 Aug 27;22:11633. doi: 10.15766/mep_2374-8265.11633 (PMC13518600; doi:10.15766/mep_2374-8265.11633)
Supplement: Supplementary file 1 [file mep_2374-8265.11633-s001.zip › F. Course Evaluation.docx]

**Appendix F. Post-Course Evaluation**

**Pediatric Orbital Cellulitis Complicated by Cavernous Sinus Thrombosis**	**Date:** ______________

1) What is your current level of training?
   a. PGY-1
   b. PGY-2
   c. PGY-3
   d. If other, please write: ____________________________________
2) How realistic did you feel the simulation experience was? (1 = very unrealistic, 6 = very realistic)

1	2	3	4	5	6

3) The debriefing session enhanced my knowledge. (1 = strongly disagree, 4 = strongly agree)

1	2	3	4

4) The knowledge I gained from the session will be helpful to me in my practice. (1 = strongly disagree, 4 = strongly agree)

1	2	3	4

5) What I learned today will help to improve patient outcomes. (1 = strongly disagree, 4 = strongly agree)

1	2	3	4

6) Overall this course was: (1 = poor, 6 = excellent)

1	2	3	4	5	6

7) My biggest take home from this course was:
