# Supplementary material for: Pediatric Orbital Cellulitis Complicated by Cavernous Sinus Thrombosis: A Pediatric Simulation Case for Residents
Source: MedEdPORTAL. 2026 Aug 27;22:11633. doi: 10.15766/mep_2374-8265.11633 (PMC13518600; doi:10.15766/mep_2374-8265.11633)
Supplement: Supplementary file 1 [file mep_2374-8265.11633-s001.zip › G. Anticipated Actions.docx]

**Appendix G.** Anticipated Actions

The following table describes actions facilitators should anticipate participants will perform during the simulation. This appendix can be used to take notes during the simulation encounter, which can later be used in the debriefing session.

| **Anticipated Action:** | **Notes:** |
| --- | --- |
| Establish roles (e.g., team leader, historian, etc.) |  |
| Demonstrate an appropriate initial approach to assessing the patient (ABCDE) |  |
| Obtain vital signs and place patient on a monitor |  |
| Identify orbital cellulitis |  |
| Initiate empiric antibiotics |  |
| Request new vital signs and new physical exam when patient exhibits clinical change |  |
| Recognize signs concerning for CST |  |
| Order CT imaging: CT brain without contrast and CT orbits with contrast |  |
| Escalate care by contacting PICU and/or surgical service, or a tertiary care center |  |
| Provide appropriate handoff to accepting physician and/or consulting team |  |
| Demonstrate effective closed loop communication |  |
